# Draft Genome Sequence of Lactobacillus helveticus Lh 23, Isolated from Natural Whey Starter

**DOI:** 10.1128/MRA.00488-20

**Published:** 2020-09-10

**Authors:** Gaia Bertani, Daniela Bassi, Claudia Cortimiglia, Monica Gatti, Pier Sandro Cocconcelli, Erasmo Neviani

**Affiliations:** a Department of Food and Drug, University of Parma, Parma, Italy; b DISTAS, Università Cattolica del Sacro Cuore, Piacenza, Italy; c Biotechnology Research Centre, Cremona, Italy; University of Arizona

## Abstract

Lactobacillus helveticus is a thermophilic lactic acid bacterium that is widely employed as a starter culture for manufacturing several Swiss and Italian hard-cooked cheeses. The sequencing of L. helveticus Lh 23, which consists of 2,100,230 bp with a GC content of 36.5%, reveals industrially useful traits and interesting metabolic pathways.

## ANNOUNCEMENT

Lactobacillus helveticus is a homofermentative, thermophilic lactic acid bacterium that plays a role as a natural dairy starter in the production of several Swiss and Italian hard-cooked cheeses due to different technological traits encoded in its chromosome (1, 2). The main technological functions of L. helveticus in the natural whey starter (NWS) culture environment are lactose depletion during curd acidification and, later, proteolytic activity over the long ripening period. We report the genome sequencing of L. helveticus Lh 23, a strain that was isolated from NWS used in the production of Grana Padano, a protected denomination of origin (PDO) Italian cheese (3). This strain, which was previously considered in a comparative study using a microarray of 38 different strains, was genetically different from the other analyzed strains with respect to genes involved in the proteolytic system and in amino acid metabolism, highlighting a high level of genotypic biodiversity due to different environmental and technological selective pressures (3–6). Therefore, a deeper knowledge of its genome may reveal industrially useful traits and interesting metabolism. Lactobacillus helveticus Lh 23 was isolated by inoculating 10-fold serial dilutions of NWS onto MRS agar (Oxoid, Waltham, MA, USA) and incubating it anaerobically at 42°C for 48 h. A single colony was picked and used for bacterial DNA extraction, which was performed using the DNeasy blood and tissue kit (Qiagen, Hilden, Germany), following the manufacturer’s instructions. DNA integrity was checked by agarose gel electrophoresis, and the concentration and purity were determined with a NanoDrop 2000 spectrophotometer (Thermo Fisher Scientific, Waltham, MA, USA).

The sample library was prepared using the Illumina TruSeq DNA sample preparation kit and was sequenced using one lane of a single flow cell on an Illumina HiSeq 1000 system with a 2 × 100-bp paired-end protocol (Genomics Platform; Parco Tecnologico Padano, Lodi, Italy), yielding 11,924,037 reads with an average insert size of 488 bp and 993.0-fold coverage. The genome was directly assembled into 312 contigs with SPAdes software v. 3.1.0; it consisted of 2,100,230 bp with a GC content of 36.5%. The draft genome contained 2,285 genes, including 1,811 protein-coding genes, 403 pseudogenes, 6 rRNAs, 62 tRNAs, and 3 other RNAs (7, 8). Gene functional annotation was performed using BLASTp with the KEGG, Swiss-Prot, and RAST v. 2.0 databases (9–11). The genome analysis revealed several genes involved in amino acid metabolism and in the proteolytic system, i.e., 128 and 39 genes, respectively. RAST annotation showed 187 genes involved in sugar metabolism, transport, and uptake, such as genes participating in lactose, galactose, maltose, fructose, chitin, and *N*-acetylglucosamine utilization. Four intact and two incomplete phage regions were identified by employing PHAge Search Tool (PHAST) (12). KEGG analysis revealed that the Lh 23 genome included 7 genes involved in the complete metabolic pathway for the biosynthesis of folate. Moreover, 2 genes involved in exopolysaccharide biosynthesis were identified through RAST annotation. Default parameters were used for all software unless otherwise specified.

### Data availability.

Data are available from the NCBI under BioProject number PRJNA310689. This whole-genome shotgun project was deposited in DDBJ/ENA/GenBank under accession number LSVJ00000000 and SRA accession number SRR12399863.
